# Maternal Perinatal Characteristics in Patients with Severe Preeclampsia: A Case-Control Nested Cohort Study

**DOI:** 10.3390/ijerph182211783

**Published:** 2021-11-10

**Authors:** Irene Aracil Moreno, Patrocinio Rodríguez-Benitez, Maria Ruiz-Minaya, Mireia Bernal Claverol, Virginia Ortega Abad, Concepción Hernández Martin, Pilar Pintado Recarte, Fátima Yllana, Cristina Oliver-Barrecheguren, Melchor Álvarez-Mon, Miguel A. Ortega, Juan A. De Leon-Luis

**Affiliations:** 1 Department of Public and Maternal and Child Health, School of Medicine, Complutense University of Madrid, 28040 Madrid, Spain; irene.aracil@salud.madrid.org (I.A.M.); prodriguezb@senefro.org (P.R.-B.); mruiz341060@salud.madrid.org (M.R.-M.); mireia.bernal@salud.madrid.org (M.B.C.); virginia.ortega@salud.madrid.org (V.O.A.); chmartin@salud.madrid.org (C.H.M.); ppintado@salud.madrid.org (P.P.R.); lourdesfatima.yllana@salud.madrid.org (F.Y.); cristina.oliver@salud.madrid.org (C.O.-B.); jaleon@ucm.es (J.A.D.L.-L.); 2 Department of Obstetrics and Gynecology, University Hospital Gregorio Marañón, 28009 Madrid, Spain; 3 Health Research Institute Gregorio Marañón, 28009 Madrid, Spain; 4 Department of Nephrology, University Hospital Gregorio Marañón, 28009 Madrid, Spain; 5 Department of Medicine and Medical Specialities, University of Alcala, 28801 Alcalá de Henares, Spain; mademons@gmail.com; 6 Ramón y Cajal Institute of Sanitary Research (IRYCIS), 28034 Madrid, Spain; 7 Immune System Diseases-Rheumatology, Oncology Service an Internal Medicine, University Hospital Príncipe de Asturias, CIBEREHD, 28806 Alcalá de Henares, Spain

**Keywords:** severe preeclampsia, maternal risk factors, pregnancy outcome

## Abstract

Preeclampsia is one of the most worrisome complications during pregnancy, affecting approximately 1 out of 20 women worldwide. Preeclampsia is mainly characterized by a sustained hypertension, proteinuria, also involving a significant organ dysfunction. Moreover, 25% of the cases could be classified as severe preeclampsia (SP), a serious condition that could be life-threatening for both the mother and fetus. Although there are many studies focusing on preeclampsia, less efforts have been made in SP, frequently limited to some specific situations. Thus, the present study aims to conduct a comparative analysis of risk factors, maternal characteristics, obstetric and neonatal outcomes and maternal complications in patients with severe preeclampsia versus patients without severe preeclampsia. Hence, 235 cases and 470 controls were evaluated and followed in our study. We described a set of variables related to the development of severe preeclampsia, including maternal age > 35 years (69.8%), gestational (26.8%) or chronic arterial hypertension (18.3%), obesity (22.6%), use of assisted reproduction techniques (12.3%), prior history of preeclampsia (10.2%) and chronic kidney disease (7.7%) All patients had severe hypertension (>160 mmHg) and some of them presented with additional complications, such as acute renal failure (51 cases), HELLP syndrome (22 cases), eclampsia (9 cases) and acute cerebrovascular accidents (3 cases). No case of maternal death was recorded, although the SP group had a higher cesarean section rate than the control group (60% vs. 20.9%) (*p* < 0.001), and there was a notably higher perinatal morbidity and mortality in these patients, who had a prematurity rate of 58.3% (*p* < 0.001) and 14 perinatal deaths, compared to 1 in the control group. Overall, our study recognized a series of factors related to the development of SP and related complications, which may be of great aid for improving the clinical management of this condition.

## 1. Introduction

Preeclampsia is a multisystem disease characterized by hypertension (HT) and proteinuria or significant organ dysfunction during the second half of pregnancy or puerperium. It is an important cause of maternal and fetal morbidity and mortality. It occurs before 37 weeks of gestation in 20% of cases, and is an important cause of iatrogenic prematurity [1,2,3].

Preeclampsia affects 4.6% (95% CI 2.7–8.2) of pregnancies according to a worldwide systematic review [4], and is two times more common in nulliparous women [5]. Other risk factors are a maternal history of preeclampsia in a previous pregnancy, chronic HT, diabetes, autoimmune diseases, obesity and chronic kidney disease. Additionally, multiple gestation, advanced maternal age and assisted reproduction techniques increase the probability of developing preeclampsia [6].

Among patients with preeclampsia, 25% meet the criteria for severe preeclampsia (SP). SP is a threat to the life of the mother and the fetus, and can lead to complications such as restricted intrauterine growth, HELLP syndrome, eclampsia, hepatic hematoma, cerebrovascular accidents, abruptio placentae or even death [1].

In the literature, there are abundant studies on preeclampsia, but most of them exclude severe forms of the disease or focus on specific conditions, such as HELLP syndrome.

The main objective of this study was to perform a comparative analysis of maternal characteristics, risk factors, obstetric and neonatal outcomes and maternal complications in patients with SP vs. patients without SP.

## 2. Materials and Methods

We carried out an observational case-control study nested in a hospital-based cohort, consisting of patients with more than 22 completed weeks of gestation who delivered at a reference center between January 2010 and December 2018. The study followed the STROBE recommendations [7].

Cases were defined as patients with suspected SP during pregnancy, childbirth or puerperium who required consultation with the nephrology service and were diagnosed with SP, according to the criteria of the American College of Obstetrics and Gynecology (ACOG) with the consensus of the obstetrics and gynecology service [1]. The criteria include SBP > 160 mmHg or DBP > 110 mmHg at two separate measurements taken at least 4 h apart and evidence of severe organ dysfunction, including neurological (headache, visual disturbances or seizures), hepatic (epigastralgia, pain in the right hypochondrium, elevated transaminases more than double the upper limit of normality), renal or hematological (thrombocytopenia, hemolysis) or the onset of acute lung edema. All patients with clinical pictures that differed from SP were excluded, including patients with HT who did not meet the severity criteria, those with nonserious organ impairment and those without associated hypertensive criteria. Similarly, patients with multiple pregnancies were excluded from the study.

Each selected case was paired with two controls using the following matching criteria: single gestation, maternal parity and seasonality, that is, patients whose delivery occurred in the 48 h before or after the case’s delivery. In this way, two groups were configured: a group of cases with SP and the group of patients who did not meet the criteria for SP, henceforth referred to as the control group. All patients belonged to the cohort of pregnant women being admitted for childbirth; therefore, the control group included both healthy pregnant women and those with mild preeclampsia or any other baseline pathology, such as chronic HT or diabetes. This ensure that the control group was as representative as possible of our real population of pregnant women. The maternal, gestational and neonatal characteristics of the controls were blinded, and the only information available was verification that they met the matching criteria and had not had SP during their current pregnancy.

For the registration of the hospital cohort and its longitudinal follow-up, a database was configured in which the variables were collected for the pregestational, gestational and perinatal period. For the SP patients, the database included a longitudinal record that was complementary to the postpartum follow-up at the nephrology service.

The following variables were collected for both groups. Maternal variables: maternal age at the time of delivery and previous parity; labor variables: onset of labor (spontaneous, induced or elective cesarean section), delivery route (vaginal or cesarean section), and days of maternal admission; neonatal variables: gestational age at the time of delivery, prematurity (GA < 37 weeks), sex of the newborn, birth weight, pH at birth, pH lower than 7 at birth, Apgar score at 1 and at 5 min of life, Apgar score at 5 min of life below 7 and perinatal mortality (between 22 completed weeks of gestation and the 7th day of life). In addition, for the SP group, the following variables were collected. Maternal variables: history of chronic arterial HT, preeclampsia in a previous pregnancy, diabetes mellitus, hypothyroidism, chronic kidney disease, chronic kidney failure, antiphospholipid syndrome and obesity. Nationality (European, African, Latin American or others) and the use of assisted reproduction techniques were also collected. Obstetric variables: diagnosis of gestational HT, gestational hypothyroidism or gestational diabetes; gestational age at the time of the SP diagnosis; presentation of preeclampsia: early-onset SP (<34 weeks) or puerperal SP (postpartum); administration of magnesium sulfate as an anticonvulsant and fetal lung maturation with betamethasone 12 mg intramuscularly (2 doses separated by 24 h). Severity variables of preeclampsia: maximum recorded systolic blood pressure (SBP) and maximum recorded diastolic blood pressure (DBP); onset of complications: HELLP syndrome, eclampsia, acute pulmonary edema, acute renal failure (ARF) (serum creatinine greater than 1.1 mg/dL), oliguria (diuresis less than 400 mL/day or <0.5 mL/kg/h), hepatic hematoma, placental abruption, cerebrovascular accident, PRESS syndrome, thrombocytopenia and need for transfusion. Neonatal variables: intrauterine growth restriction (IGR), defined as estimated fetal weight (EFW) lower than the 3rd percentile for gestational age and sex or lower than the 10th percentile with alterations in the fetal Doppler study and the need for admission to the neonatal intensive care unit (NICU) after delivery. Postpartum maternal variables: presence of HT at hospital discharge and number of antihypertensive drugs necessary for blood pressure control, ARF at discharge and 3 months after delivery, presence of proteinuria 3 months after delivery and maternal loss to follow-up. Maternal laboratory variables: determination of plasma creatinine, 24 h urine creatinine clearance and proteinuria at the time of SP diagnosis, at hospital discharge and at 12 weeks after delivery; determination of uric acid, transaminase (GPT) and platelet levels at the time of diagnosis and at hospital discharge and maximum recorded level of lactase dehydrogenase (LDH) and postpartum hemoglobin. Regarding the statistical analysis, the distribution of the variables in the case-control study was analyzed, the results were compared and a descriptive analysis (as a case series) of the variables that were collected only for the pregnant women with SP was performed.

Data compiled for the study were entered in a Microsoft Office Excel database, version 16.42 (Microsoft, Redmond, WA, USA) and statistical analysis of data was performed using the package SPSS version 21 software (IBM Co., Somers, NY, USA). An analytical study was conducted comparing the main maternal characteristics, laboratory test results, obstetric and perinatal characteristic and maternal/neonatal mortality and morbidity data between the two groups. Significance was set at *p* < 0.05. Quantitative variables were expressed as the mean ± standard deviation (SD) and categorical variables as number of patients and rates (%). Univariate analysis was performed using Fisher’s exact test, chi-squared test or Student’s *t*-test, as appropriate.

The study protocol was presented to and authorized by the Ethics Committee for Medical Research of our center.

## 3. Results

**Figure 1:**
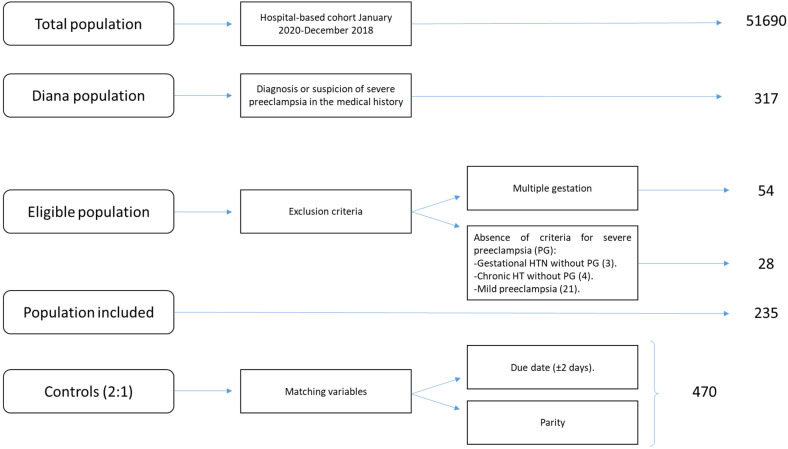
Flowchart of the nested case-control study in a hospital-based cohort.

During the study period (January 2010 to December 2018), 51,690 women were assisted at our center, which performs an average of 5743 deliveries/year; of these women, 235 (0.45%) had SP. Figure 1 presents the flowchart of the patients according to the inclusion and exclusion criteria; finally, 235 cases and 470 controls (705 patients total) were obtained from this hospital-based cohort. It should be noted that 73.2% of the consultations between the obstetrics and nephrology services were for SP, and that 21 patients with this initial diagnosis did not meet the current criteria for severity, which have changed over time, and were therefore discarded from the study.

Table 1 presents the distribution of maternal, intrapartum and neonatal variables for all patients and by group, along with the degree of significance. There were no cases of maternal mortality in any of the groups. Among the study variables, no differences were found between the two groups in terms of maternal age, which had an average of 33 years, or fetal sex; however, the rest of the variables showed statistically significant differences. The clinical differences in the percentage of both elective and all-cause (global) cesarean sections stand out, the latter of which was three times higher in the case series (60% vs. 20.9%; *p* < 0.001). Among the cases, the newborns’ gestational age at birth and weight were lower, while the percentages of newborns with a pH less than seven at birth and an Apgar score less than seven at 5 min after delivery were significantly higher. The hospital stays of the cases were 5 days longer on average. Finally, a total of 15 perinatal deaths occurred during the study period: 14 in the SP group, 10 of which were intrauterine, and 1 in the control group, which was also intrauterine.

Table 2 presents the distribution of the complementary variables in the assessment and follow-up of the SP cases up to 12 weeks postpartum. Regarding maternal variables, 10.2% (24/235) had experienced preeclampsia in previous pregnancies, a percentage that increased to 19.5% when we excluded the 112 participants who were primiparous (24/123).

In addition, 18.3% of the patients were chronically hypertensive, 1 in 5 was obese (BMI > 30) and 12.3% had undergone in vitro fertilization treatment. Regarding the obstetric variables, 38.7% of the cases had early onset SP before 34 weeks of gestation, with a mean gestational age at diagnosis of 34 weeks, while 22.6% of cases occurred in the postpartum and puerperium periods. Overall, two out of three patients received treatment with magnesium sulfate. Regarding the severity features of preeclampsia, the most frequent complication was acute renal failure (21.7%), followed by thrombocytopenia (14.5%) and HELLP syndrome (9.4%). Other complications were eclampsia (3.8%), placental abruption (1.3%) and acute lung edema (1.3%). Among the neonatal features, the incidence of IGR was 23.8%, and 28.7% of these newborns required admission to the NICU. Finally, after being discharged from the hospital after an average stay of 10 days, the vast majority of the patients continued antihypertensive treatment with at least one drug. After hospital discharge, 11.7% of all cases were lost to follow-up.

Table 3 presents the comparative analysis of the maternal, hematological and biochemical variables at diagnosis, hospital discharge and 12 weeks after delivery. At the time of diagnosis, the mean proteinuria, uric acid and transaminase levels were higher than normal, while the mean platelet count was slightly decreased. When the variables were compared, significant differences between the different study times were found for all variables, with the exception of creatinine clearance.

## 4. Discussion

During the study period, a total of 235 patients of SP were admitted within a cohort of 51,690 pregnant women who delivered at our center, which represents an incidence of 0.45% of all deliveries and an average of 26 cases per year. The comparative analysis of cases and controls showed significant differences in almost all of the maternal-perinatal variables analyzed. In the group of patients with SP, a high prevalence of pregestational and obstetric risk variables related to the development of preeclampsia stands out, particularly maternal age over 35 years (69.8%), chronic arterial (18.3%) or gestational HT (26.8%), history of preeclampsia in a previous pregnancy (10.2%), obesity (22.6%), chronic kidney disease (7.7%) and the use of assisted reproduction techniques (12.3%). Regarding the criteria used to define the severity of the condition, all patients had severe HT (> 160 mmHg); in addition, there were 22 cases of HELLP syndrome, 9 cases of eclampsia, 3 acute cerebrovascular accidents and 51 patients with acute renal failure. No case of maternal death was recorded in any of the study groups, although, as previously mentioned, the case group had greater maternal morbidity, and 35% of the patients had at least one severe complication related to SP. In addition, the SP group had a higher cesarean section rate than the control group (60% vs. 20.9%) (*p* < 0.001), and there was a notably higher perinatal morbidity and mortality in patients with SP, who had a prematurity rate of 58.3% (*p* < 0.001) and 14 perinatal deaths, compared to one in the control group. Additionally, in most cases, the analyzed parameters presented alterations at the time of diagnosis and progressively normalized during hospitalization; this can be clearly seen in the case of proteinuria, which showed a decrease in mean levels from 2.82 g/day at diagnosis to 0.97 g/day at the time of hospital discharge and 0.14 g/dL 3 months later.

In comparison with our SP incidence of 0.45%, Sibai et al. [8] found an incidence of 0.48%, with a total of 1153 cases of SP in five US states between 1977 and 1985. Liu et al. [9] found an SP incidence of 0.65% at a center in Taiwan between 1994 and 2003. However, more recent studies at the single-center level describe a higher incidence of this condition; Turgut et al. [10] reported an incidence of 1.41% in Turkey between 2005 and 2008 and Ngwenya et al. [11] reported an incidence of 1.3% in Zimbabwe between 2016 and 2018. These differences are probably due to geographical, temporal, ethnic and socioeconomic factors. It is worth mentioning that during the study period, a progressive increase in the annual number of cases was observed, especially in 2018, where an incidence of 1.2% was noted. This may be due to diagnostic improvements and the establishment of multidisciplinary protocols through close collaboration between the nephrology and obstetrics departments, but it is also likely that the number of cases was underestimated in the first years of the study.

Regarding maternal characteristics, it is noteworthy that in both groups, the mean maternal age was 33 years, higher than that found in other similar studies [9,12,13,14]. However, the percentage of women over 35 and 40 years (69.8% and 28.9%, respectively) was twice as high in the SP group than in the control group. This proportion of pregnant women of advanced age was much higher than that described by Liu et al. [9] in 2008 in Taiwan; in that study, only 28.3% of patients with SP or gestational HT were older than 35 years, and 9.1% were older than 40 years, which are lower values than those we found in our controls. In our opinion, these differences may be due to differences in demographic, cultural and temporal factors between studies, but it is clear that advanced age poses an added risk for the development of preeclampsia [9,12,13,14].

Regarding the delivery route, 60% of the patients with SP underwent a cesarean section, a percentage that was significantly higher than that of the controls. The percentage of cesarean sections reported in other studies varies from 58.8%, found by Yucesoy et al. [12], to 88.9%, found by Jantasing et al. [13]. This difference between cases and controls is explained by the need to end pregnancy urgently in the face of clinical-analytical worsening of the maternal condition or deterioration of the fetal state, due to the associated placental insufficiency and the consequent risk of loss of fetal well-being.

Another significant difference found in our study was a longer duration of hospital stay; we found a mean admission duration of 9.7 ± 6.1 days, a figure that is much lower than the 11.2 ± 17.9 days described by Turgut et al. [10]. This difference is probably influenced by the higher incidence of cesarean sections, but also by the need for patient control and monitoring, sometimes for more than 2 weeks.

Regarding perinatal characteristics, it is noteworthy that the average gestational age at birth of the children born to mothers with SP was 34.77 ± 3.97 weeks, 4 weeks lower than that found in the controls and similar to that of other series of patients with SP [9,10]. This is because the rate of prematurity was five times higher in the SP patients (58.3% vs. 9.6%), which is slightly higher than the prematurity rate of 55.6% found by Li et al. [14] among 1396 women with preeclampsia. In correlation with this decrease in the average gestational age, the newborns had an average birth weight of 2218 ± 905 g, 1 kg less than the average weight of the control group (*p* < 0.001). This low birth weight was also influenced by the prevalence of IGR in this group, which in our series was 23.8%, close to the 25% found in the study by Kongwattanakul et al. [15]. SP is one of the main known causes of IGR and has an OR of 2.16 (95% CI, *p* = 0.026) according to Liu et al. Both pathologies originate from anomalous placentation, which conditions increased resistance to uteroplacental flow and placental insufficiency secondary to the endothelial destruction of a low-resistance tissue subjected to high pressure (Kwiatkowski) [16].

Both circumstances, IGR and prematurity, are the factors with the greatest influence on the other studied perinatal and neonatal variables. Most guidelines recommended that in patients with SP, gestation should be ended after 34 weeks of pregnancy, although there is debate about the benefit of waiting until 37 weeks in stable patients [17,18,19] and when the maternal and/or fetal situation does not allow expectant management. Based on this, we can say that the mean time between the diagnosis of SP and delivery was 2.6 days, which leads to a tendency toward early termination of pregnancy in these cases in our center. Turgut et al. [10] reported a mean time of between 0.7 and 1.5 days, and Liu et al. [9] reported a mean time of up to 4.4 days.

The direct impact of prematurity and placental insufficiency associated with SP on perinatal outcomes was an increase in perinatal morbidity and mortality (Table 1). In the case group, there was a higher percentage of pathological results for the umbilical cord blood pH and the Apgar score, which was lower than seven at 5 min after delivery in 9.4% in the cases compared to 0.4% of the matched controls. In the literature, the incidence of pathological Apgar scores varies between 7.1% and 32% [10,11,13]. A total of 28.5% of newborns born to mothers with SP required admission to the NICU, slightly lower than the 31.4% who were admitted in the study by Yucesoy et al. [12].

Regarding perinatal mortality, of the 15 perinatal deaths in the present study, 10 were intrauterine and 5 occurred in the first 7 days of life. Among the cases, there were 14 deaths (93.3% of total mortality), and the rate of perinatal mortality was 30 times higher in the SP group (6% vs. 0.2%). In comparison, Turgut et al. [10] reported a 10.7% incidence of neonatal and intrauterine mortality in 467 women with SP, Jantasing et al. reported a rate of 11.1% in patients with early onset SP (3.9% in newborns with a gestational age greater than 28 weeks and 36.4% in those with a gestational age below 28 weeks), and Ngwenya et al. [11] reported an early neonatal mortality rate of 22.8% in 378 women with SP in Zimbabwe, taking into account that 26.7% of them delivered before 30 weeks of gestational age.

In addition to the comparison of cases and controls, a series of variables of special interest in patients with SP was analyzed, including risk factors for the development of the disease and related complications (Table 3). Specifically, among the risk factors associated with the development of SP, 10.2% of the cases had developed preeclampsia in previous pregnancies and 18.3% had chronic HT. These findings were similar to the results of Jantasing et al. [13] in 99 patients with severe early preeclampsia, 14.1% of whom had previously had preeclampsia and 16.2% of whom had chronic HT, but were much lower than those reported in the study of Guerrier et al. [20], in which 26% of patients with SP had chronic HT. Other prevalent risk factors in our study were obesity (22.6%), IVF use (12.3%) and hypothyroidism (10.2%); these values were higher than the 9.3% rate of IVF use and the 4.1% incidence of thyroid pathology described by Li et al. in 1396 patients with SP [14]. All cases had proteinuria and high blood pressure in the severe range. The maximum systolic and diastolic BP was 180 and 104 mmHg, respectively, similar to the values described in similar studies [10,13,14].

Among the recorded maternal complications, the most common was acute renal failure (21.7%), followed by thrombocytopenia (14.5%), HELLP syndrome (9.4%), oliguria (7.2%) and eclampsia (3.8%). The incidence of ARF was similar to that described by Jantasing et al. (18.1%), but much higher than that described by Turgut et al. [10] (2.4%) although their incidence of oliguria was 40%, and that described by Nankali et al. [21] (2.3%). These striking differences may be due to differences in diagnostic criteria, since in our study, ARF was defined as plasma creatinine (Cr) > 1.1 mg/dL, while Turgut et al. established stricter criteria of Cr > 2 mg/dL, oliguria/anuria and decreased creatinine clearance. Of the patients with a diagnosis of ARF, the creatinine level corrected spontaneously after delivery, and ARF persisted in only 3.8% at hospital discharge and only three patients at 3 months after delivery, all of whom had chronic renal insufficiency prior to pregnancy. In contrast, the incidence of eclampsia and placental abruption was lower than that reported in similar studies [9,10]. During the study, three acute cerebrovascular accidents occurred among the cases: one patient had a stroke 30 days after delivery, another had parietal sinus thrombosis and another had hemorrhage in the basal ganglia during pregnancy. There was also a case of posterior reversible encephalopathy (PRESS syndrome).

Regarding maternal mortality, it is noteworthy that despite these serious complications, there were no maternal deaths in our case series; however, in the literature, maternal mortality has been reported in up to 1.17% of pregnant women with SP [9,12,13]. Our low incidence is probably due to the early termination of pregnancy in these cases and to the management of patients with severe complications by experienced intensive care specialists.

Finally, with respect to the analytical alterations in patients with SP (Table 3), we found that at the time of diagnosis, the mean plasma creatinine was 0.87 mg/dL, a value very similar to that of the SP patients in the study by Turgut et al. [10]. However, the patients in that study had a mean daily proteinuria level of 7.6 ± 11.2 g, much higher than we found; additionally, the levels of ALT (36 ± 30.7 IU/L) and uric acid (5, 9 ± 1.5 mg/dL) and the platelet counts (222,636 ± 77,574.5/µcL) prior to delivery were much less altered than our results. This may be because in our series, we included patients with HELLP syndrome, a group that was analyzed separately in the Turgut study. These analytical parameters were also analyzed at the time of hospital discharge, at which point the majority had already returned to within the normal range; the exception was uric acid, for which the mean persisted at greater than 5 mg/dL. In the subsequent follow-up, the mean proteinuria level continued to decrease after hospital discharge (0.97 g/24 h) until 3 months later (0.14 g/24 h), while plasma creatinine decreased initially but remained stable after discharge.

In terms of the strengths of the study, the large number of patients included and the multidisciplinary approach to the diagnosis, treatment and control of cases should be highlighted. An additional strength was the nested cohort case-control methodology, in which the cohort represented the total number of patients who delivered at a single center (more than 50,000 laboring women). It is also worth highlighting the matching of the groups according to maternal parity and delivery date, which prevents the confounding influence of nulliparity and variations in the clinical management of this pathology over the years. In terms of limitations, those associated with retrospective studies are worth mentioning, including the possible underestimation of the number of cases in previous years, as mentioned at the beginning of the discussion section.

This may have contributed to the striking increase in the number of recorded cases of SP, which was 2–3 times higher in recent years than in the first years of the study, and although it was not a priority of this study, future research should take this temporal factor into account.

## 5. Conclusions

SP is a pathology that affects approximately 1 in 220 pregnant women in our context, but despite its low incidence, it is a leading cause of maternal and neonatal morbidity and mortality worldwide. It is associated with a considerable increase in the cesarean section rate and with the occurrence of serious maternal complications, including cerebrovascular accidents, eclampsia and acute renal failure, among others, which were present in 35% of our cases. From the neonatal point of view, iatrogenic prematurity associated with placental alterations and IGR, among other factors, led to worse neonatal outcomes, including a NICU admission rate of 28.5% and a perinatal mortality rate 30 times higher than that of our general population of pregnant women, represented by the controls. These results indicate the importance of early detection to ensure that antihypertensive and neuroprotective treatment is established as soon as possible, allowing for fetal lung maturation and terminating the pregnancy when necessary to prevent the occurrence of serious complications. Care for this group of patients should be provided at tertiary centers by an experienced multidisciplinary team of specialists in maternal and neonatal intensive care.

## Figures and Tables

**Table 1:** Comparative analysis of the distribution of clinical variables in all pregnant women, as well as between cases of severe preeclampsia (SPG) and their controls. Variables are expressed by mean ± SD and n (%).

	Overall	Cases (SP)	Controls	*p* Value
N, %	705	235 (33.3%)	470 (66.7%)	
Maternal features:				
Maternal age at birth, mean ± SD	33.62 ± 5.88	33.50 ± 6.32	33.69 ± 5.65	0.78
Maternal age > 35 years, n, %	334 (47.4%)	164 (69.8%)	170 (36.2%)	<0.001
Maternal age > 40 years, n, %	131 (18.6%)	68 (28.9%)	63 (13.4%)	<0.001
Nuliparous, n, %	341 (48.3%)	112 (47.7%)	229 (48.7%)	0.67
Delivery features:				
Elective C-Section, n, %	113 (16%)	95 (40.4%)	18 (3.8%)	<0.001
Global C-Section, n, %	239 (33.9%)	141 (60%)	98 (20.9%)	<0.001
Days of maternal admission, mean ± SD	5.64 ± 4.7	9.73 ± 6.09	3.58± 1.39	<0.001
Maternal mortality, n, %	0	0	0	
Neonatal features:				
Gestational age at delivery (weeks), mean ± SD	37.41 ± 3.36	34.77 ± 3.97	38.74 ± 1.95	<0.001
Prematurity, n, %	179 (25.8%)	137 (58.3%)	45 (9.6%)	<0.001
Male, n, %	359 (50.9%)	108 (46%)	251 (53.4%)	0.62
Birth weight, mean ± SD	2879 ± 833	2218± 905	3210± 552	<0.001
pH at birth, mean ± SD	7.26 ± 0.08	7.23 ± 0.09	7.27 ± 0.08	<0.001
Unknown pH, mean ± SD	37 (5.2%)	20 (8.5%)	17 (3.6%)	
Apgar at 1st minute, mean ± SD	8.17 ± 1.72	7.18 ± 2.31	8.65 ± 1.13	<0.001
Apgar at 5 min, mean ± SD	9.28 ± 1.52	8.55 ± 2.16	9.64 ± 0.88	<0.001
Apgar Unknown n, %	4 (0.5%)	3 (1.2%)	0 (0%)	
Perinatal morbidity and mortality				
pH < 7 at birth, n, %	14 (1.9%)	12 (5.1%)	2 (0.4%)	<0.001
Apgar at 5 min < 7, n, %	27 (3.8%)	22 (9.4%)	5 (1.1%)	<0.001
Perinatal mortality, n, %	15 (2.1%)	14 (6%)	1 (0.2%)	<0.001
Intrauterine mortality, n, %	10 (1.4%)	9 (3.8%)	1 (0.2%)	<0.001

**Table 2:** Description of the clinical variables in cases of severe preeclampsia. Variables are expressed by mean ± SD and n (%). PA = personal history, HT = arterial hypertension, BMI = body mass index, IVF = in vitro fertilization, GA = gestational age, NICU = neonatal intensive care unit.

	Overall n = 235
Maternal features	
Chronic hypertension, n,%	43 (18.3%)
Previous preeclampsia, n,%	24 (10.2%)
Diabetes Mellitus, n,%	6 (2.6%)
Chronic hypothyroidism, n,%	24 (10.2%)
Chronic kidney disease, n,%	18 (7.7%)
Chronic renal failure (GFR < 60 mL/min/1.73^2^), n,%	3 (1.3%)
Antiphospholipid syndrome, n, %	6 (2.6%)
BMI > 30	53 (22.6%)
Nationality, n,%	
European	140 (59.6%)
African	13 (5.5%)
Latin American	78 (33.2%)
Other	4 (1.7%)
IVF, n,%	29 (12.3%)
Obstetric features:	
Gestational hypertension, n,%	63 (26.8%)
Gestational hypothyroidism, n,%	25 (10.6%)
Gestational diabetes, n,%	20 (8.5%)
GA at diagnosis of severe preeclampsia (weeks), mean ± SD	34.40 ± 4.46
Form of presentation of severe preeclampsia, n,%	
Early onset (GA < 34 weeks)	91 (38.7%)
Puerperal preeclampsia	53 (22.6%)
Magnesium Sulfate, n,%	158 (67.2%)
Lung maturation with corticosteroid therapy, n,%	105 (44.7%)
PE Severity features	
Maximum systolic blood pressure (mmHg), mean ± SD	180 ± 16
Maximum diastolic blood pressure (mmHg), mean ± SD	104 ± 12
HELLP syndrome, n,%	22 (9.4%)
Eclampsia, n,%	9 (3.8%)
Acute lung edema, n,%	3 (1.3%)
Acute renal failure (plasma creatinine > 1.1 mg/dL), n,%	51 (21.7%)
Oliguria (<400 mL/day or <0.5 mL/kg / h), n,%	17 (7.2%)
Hematoma of the liver, n,%	2 (0.9%)
Abruptio placentae, n,%	3 (1.3%)
Acute cerebrovascular accident, n,%	3 (1.3%)
PRESS syndrome, n,%	1 (0.2%)
Thrombocytopenia (<100,000 platelets), n,%	34 (14.5%)
Need for transfusion of packed red blood cells, n,%	21 (8.9%)
Neonatal Features	
Restricted intrauterine growth (CIR), n,%	56 (23.8%)
Newborn admission to NICU, n,%	67 (28.5%)
Postpartum features:	
HBP at discharge, n,%	227 (96.6%)
Number of antihypertensive drugs at discharge, n,%	
No antihypertensive drug	8 (3.4%)
1 antihypertensive drug	97 (41.3%)
2 antihypertensive drugs	109 (46.4%)
3 or more antihypertensive drugs	21 (8.9%)
Renal failure at discharge, n,%	9 (3.8%)
Persistence of renal failure 12 weeks after delivery, n,%	3/9 (33.3%)
Positive proteinuria at 12 weeks, n,%	167 (71.1%)
Postpartum hemoglobin (g/dL, mean ± SD)	10.76 ± 1.74
Loss to follow-up after hospital discharge, n,%	27 (11.5%)

**Table 3:** Temporal evolution of laboratory parameters in cases of severe preeclampsia (PS) at the time of diagnosis, at hospital discharge and at 12 weeks after delivery. GPT = glutamic pyruvic transaminase; ALT = alanine aminotransferase; LDH = lactate dehydrogenase. * *p* < 0.001 between diagnosis and discharge, ǂ *p* < 0.001 between diagnosis and 12 weeks postpartum, ^®^ was been significant and had only been performed in 69 patients.

	Diagnosis	Discharge	*p*	At 12 Weeks	*p*
Plasma creatinine (mg/dL), mean ± SD	0.87 ± 0.55	0.67 ± 0.25	*	0.68 ± 0.19	ǂ
24 h urine creatinine clearance (ml/min), mean ± SD	113.14 ± 41.96	117.37 ± 33.85		113.03 ± 31.44	^®^
Proteinuria at diagnosis of PE (gr/day), mean ± SD	2.82 ± 2.92	0.97 ± 1.057	*	0.14 ± 0.26	ǂ
Uric acid at diagnosis of PE (mg/dL), mean ± SD	7.24 ± 1.76	5.71± 1.45	*		
GPT (ALT) at diagnosis of PE (IU/L), mean ± SD	61.96 ± 116.03	29.12 ± 24.22	*		
Platelets/µcL at diagnosis of PE, mean ± SD	167,485 ± 72,230	287,536 ± 100,015	*		
Peripartum LDH (IU/L), mean ± SD	344.97 ± 300.83	240.12 ± 64.61	*		

## Data Availability

Data from this study are available at the obstetrics service of the Hospital General Universitario Gregorio Marañón in Madrid and will be made available upon request.

## References

[1] American College of Obstetricians and Gynecologists Committee on Practice Bulletins (2020). Gestational Hypertension and Preeclampsia. Obstet. Gynecol..

[2] Payne B., Magee L.A., von Dadelszen P. (2011). Assessment, surveillance and prognosis in pre-eclampsia. Best Pr. Res. Clin. Obs. Gynaecol..

[3] Magee L.A., Pels A., Helewa M., Rey E., von Dadelszen P., Audibert F., Bujold E., Côté A.-M., Douglas M.J., Eastabrook G. (2014). Diagnosis, evaluation, and management of the hypertensive disorders of pregnancy: Executive summary. J. Obs. Gynaecol. Can..

[4] Abalos E., Cuesta C., Grosso A.L., Chou D., Say L. (2013). Global and regional estimates of preeclampsia and eclampsia: A systematic review. Eur. J. Obs. Gynecol. Reprod. Biol..

[5] Ananth C.V., Keyes K.M., Wapner R.J. (2013). Pre-eclampsia rates in the United States, 1980–2010: Age-period-cohort analysis. BMJ.

[6] Bartch E., Medcalf K.E., Park A.L., Ray J.G. (2016). Clinical Risk factors for preeclampsia determined in early pregnancy: Systematic review and meta-analysis of large cohort studies. BMJ..

[7] Von Elm E., Altman D.G., Egger M., Pocock S.J., Gøtzsche P.C., Vandenbroucke J.P. (2008). The Strengthening the Reporting of Observational Studies in Epidemiology [STROBE] statement: Guidelines for reporting observational studies. Gac. Sanit..

[8] Sibai B.M., Taslimi M.M., El-Nazer A., Amon E., Mabie B.C., Ryan G.M. (1986). Maternal-perinatal outcome associated with the syndrome of hemolysis, elevated liver enzymes, and low platelets in severe preeclampsia-eclampsia. Am. J. Obs. Gynecol..

[9] Liu C.M., Cheng P.J., Chang S.D. (2008). Maternal Complications and Perinatal Outcomes Associated with Gestational Hypertension and Severe Preeclampsia in Taiwanese Women. J. Med. Assoc..

[10] Turgut A., Demirci O., Demirci E., Uludoğan M. (2010). Comparison of maternal and neonatal outcomes in women with HELLP syndrome and women with severe preeclampsia without HELLP syndrome. J. Prenat. Med..

[11] Ngwenya S., Jones B., Mwembe D. (2019). Determinants of adverse maternal and perinatal outcomes in severe preeclampsia and eclampsia in a low-resource setting, Mpilo Central Hospital, Bulawayo, Zimbabwe. BMC Res. Notes.

[12] Yücesoy G., Özkan S., Bodur H., Tan T., Çalışkan E., Vural B., Çorakçı A. (2005). Maternal and perinatal outcome in pregnancies complicated with hypertensive disorder of pregnancy: A seven year experience of a tertiary care center. Arch. Gynecol. Obs..

[13] Jantasing S., Tanawattanacharoen S. (2008). Perinatal Outcomes in Severe Preeclamptic Women between 24-33^+6^ Weeks’ Gestation. J. Med. Assoc. Thai..

[14] Li X., Zhang W., Lin J., Liu H., Yang Z., Teng Y., Duan S., Lin X., Xie Y., Li Y. (2018). Risk factors for adverse maternal and perinatal outcomes in women with preeclampsia: Analysis of 1396 cases. J. Clin. Hypertens..

[15] Kongwattanakul K., Saksiriwuttho P., Chaiyarach S., Thepsuthammarat K. (2018). Incidence, characteristics, maternal complications, and perinatal outcomes associated with preeclampsia with severe features and HELLP síndrome. Int. J. Women’s Health.

[16] Kwiatkowski S., Dołegowska B., Kwiatkowska E., Rzepka R., Marczuk N., Loj B., Torbè A. (2017). Maternal endothelial damage as a disorder shared by early preeclampsia, late preeclampsia and intrauterine growth restriction. J. Perinat. Med..

[17] Chappell L.C., Brocklehurst P., Green M.E., Hunter R., Hardy P., Juszczak E., Linsell L., Chiocchia V., Greenland M., Placzek A. (2019). Planned early delivery or expectant management for late preterm pre-eclampsia (PHOENIX): A randomised controlled trial. Lancet.

[18] Magee L.A., Yong P.J., Espinosa V., Côté A.M., Chen I., Von Dadelszen P. (2009). Expectant Management of Severe Preeclampsia Remote from Term: A Structured Systematic Review. Hypertens. Pregnancy.

[19] Haddad B., Sibai B.M. (2009). Expectant management in pregnancies with severe pre-eclampsia. Semin. Perinatol..

[20] Guerrier G., Oluyide B., Keramarou M., Grais R.F. (2013). Factors associated with severe preeclampsia and eclampsia in Jahun, Nigeria. Int. J. Womens Health.

[21] Nankali A., Malek-Khosravi S.H., Zangeneh M., Rezaei M., Hemati Z., Kohzadi M. (2013). Maternal Complications Associated with Severe Preeclampsia. J. Obstet. Gynecol. India.

